# Beneficial subgroups for PD-1 inhibitor plus chemotherapy in first-line treatment of advanced esophageal squamous cell carcinoma: A systematic review and meta-analysis

**DOI:** 10.1097/MD.0000000000047981

**Published:** 2026-03-20

**Authors:** Rui Gao, Dong Wang, Lili Su, Xiangyu Zhang, Tingting Dai

**Affiliations:** a Department of Pharmacy, Tianjin Cancer Hospital Airport Hospital, Tianjin, China; b National Clinical Research Center for Cancer, Key Laboratory of Cancer Prevention and Therapy, Tianjin’s Clinical Research Center for Cancer, Tianjin Medical University Cancer Institute and Hospital, Tianjin, China; c Department of Pharmacy, Tianjin Medical University Cancer Institute and Hospital, Tianjin, China.

**Keywords:** esophageal squamous cell carcinoma, first-line treatment, meta-analysis, programmed death-1 antibody, programmed death-ligand 1

## Abstract

**Background:**

Esophageal cancer exhibits peak incidence in Asia and Africa, representing the sixth most common malignancy and seventh leading cause of global cancer mortality. Esophageal squamous cell carcinoma (ESCC) constitutes 90% of esophageal cancer cases. The European Medicines Agency approved programmed death 1 (PD-1) inhibitors plus chemotherapy as a first-line treatment for high PD-1-expressing ESCC.

**Methods:**

We systematically searched randomized controlled trials of PD-1 or PD-L1 inhibitors as first-line treatment from PubMed, Embase, and Cochrane Library. The following outcomes were combined: overall survival, progression-free survival, objective response rate, and treatment-related adverse events (TRAEs). Bias risk was rigorously evaluated using the Cochrane Risk of Bias Tool. RevMan 5.3 and R Studio (Boston) were utilized for data synthesis in this meta-analysis, with sensitivity analyses comparing fixed- and random-effects models to reinforce findings.

**Results:**

A total of 4702 patients (PD-1 inhibitors plus chemotherapy: 2529; chemotherapy: 2173) were enrolled in 8 randomized controlled trials. Compared with conventional chemotherapy, first-line PD-1 inhibitors plus chemotherapy significantly improved the overall survival (hazard ratio = 0.68, 95% confidence interval (CI): 0.63–0.74; *P* < .00001) and objective response rate (relative risk [RR] = 2.03, 95% CI: 1.80–2.29; *P* < .00001) of advanced ESCC patients. Moreover, PD-1 inhibitor-based therapy provided benefits in progression-free survival (hazard ratio = 0.62, 95% CI: 0.58–0.66; *P* < .00001). But PD-1 inhibitors were not associated with statistically lower incidences of TRAEs and grade 3 to 5 TRAEs. In subgroup analyses, except the limited benefit observed in the programmed death-ligand 1 (PD-L1) combined positive score < 1 subgroup, none of the following factors significantly influenced the efficacy of PD-1 inhibitor therapy: advanced age, metastatic status, number of metastatic organs, or presence of liver metastases.

**Conclusion:**

The combination of PD-1 inhibitors with chemotherapy demonstrates superior efficacy as first-line therapy for advanced esophageal squamous cell carcinoma. Both elderly patients and those with metastatic involvement derive universal benefit without increased adverse risks. However, patients with PD-L1 combined positive score < 1 may experience restricted clinical benefits. Thus, more precise predictive markers are required to stratify potential responders, enabling broader patient populations to derive benefits from PD-1 inhibitor-chemotherapy regimens.

## 1. Introduction

Esophageal cancer (EC) is one of the most prevalent malignant tumors worldwide.^[1]^ The 2 primary histologic subtypes of EC are esophageal squamous cell carcinoma (ESCC) and adenocarcinoma, with ESCC notably accounting for 90% of EC cases in Asia.^[2]^ ESCC can occur throughout the entire length of the esophagus and is more difficult to treat than esophageal adenocarcinoma, which is prevalent in western populations. Furthermore, since many patients are diagnosed at advanced stages, posing significant challenges for disease management, only with a 15% to 25% 5-year survival rate.^[3,4]^ Additionally, platinum plus paclitaxel/fluorouracil chemotherapy has been the standard first-line therapy for patients with advanced or metastatic ESCC for the past few decades despite a limited median overall survival (OS) of 12 months. When breakthrough to first-line therapy for advanced ESCC, taxane or irinotecan monotherapy is frequently used. Therefore, there is a pressing need for novel treatment plans to enhance the efficacy of therapy.^[5]^

In recent years, immune checkpoint inhibitors, in particular programmed cell death-1/ligand-1 (PD-1/PD-L1) inhibitors, have made significant advances in cancer therapy.^[6–8]^ PD-1 inhibitors are increasingly being approved by the National Comprehensive Cancer Network (NCCN) and Chinese Society of Clinical Oncology (CSCO) guidelines for front-line treatment of various cancers, including ESCC. While the updated NCCN guidelines have specified requirements for PD-L1 expression levels in using PD-1 inhibitors, it remains debatable whether PD-L1-negative patients can benefit from PD-1 inhibitor combination chemotherapy. In previous studies, a growing number of randomized controlled trials (RCTs) have focused on PD-1 inhibitors as first-line agents beyond chemotherapy for the treatment of advanced ESCC.^[9-16]^ Since pembrolizumab became the first PD-1 inhibitor approved globally and in China for first-line treatment of advanced EC in 2021, a growing number of PD-1 inhibitors have entered the therapeutic landscape for first-line advanced ESCC, other PD-1 inhibitors are also underway. Therefore, we conducted an updated meta-analysis on published data to confirm the efficacy and safety of PD-1 inhibitors for the treatment of advanced ESCC. Subgroup analyses based on patient age, metastatic status, and PD-1 expression levels were also conducted to determine whether patient population can benefit from the treatment.

## 2. Methods

### 2.1. Search strategy

From inception to October 2024, an extensive literature search was conducted using PubMed, Embase, Cochrane, and the unpublished abstract and poster databases of the American Society of Clinical Oncology (ASCO) and European Society for Medical Oncology (ESMO), with English restrictions. References of relevant articles were also manually examined to avoid lapses in the relevant articles. The following terms: (Esophageal squamous cell carcinoma OR esophageal Squamous Cell Carcinoma OR esophageal squamous carcinoma OR esophageal squamous cell carcinomas squamous cell carcinoma of esophagus) AND (anti-PD-1 OR anti-PD-1 antibody OR anti-PD-1 mAb OR anti-PD-1 monoclonal antibody OR immunotherapy OR nivolumab OR opdivo OR ONO-4538) AND chemotherapy AND (randomized controlled trial OR randomized OR placebo). The search strategy was evaluated independently by 2 authors according to the Preferred Reporting Items for Systematic review and Meta-Analysis (PRISMA).

### 2.2. Inclusion and exclusion criteria

Studies meeting the inclusion criteria were: RCTs involving patients with advanced/metastatic ESCC receiving first-line therapy. Eligible trials employed a comparative design of anti-PD-1 inhibitors combined with chemotherapy versus conventional chemotherapy alone, with at least 3 prespecified endpoints from the following: OS, progression-free survival (PFS), objective response rate (ORR), treatment-related adverse events (TRAEs), or grade 3 to 5 TRAEs.

Exclusion criteria comprised: duplicate publications (only the most comprehensive dataset was retained); phase I/II trials; studies lacking extractable data. Any discrepancies in study selection were resolved through consensus among 3 independent reviewers.

### 2.3. Data extraction and quality assessment

First, the name of the clinical trial, year of publication, name of the first author, and the total number of participants were taken from the study’s information. Second, the following survival outcomes were gathered: OS, PFS, ORR, TRAEs, and grade 3 to 5 TRAEs. Finally, for subgroup analysis, age, PD-L1 status, disease status and several TRAEs were retrieved. Cochrane risk of bias tool was used to conduct the risk assessment. Sensitivity analysis was used to evaluate the robustness and dependability of results. Two independent authors extracted the data and judged the quality.

### 2.4. Statistical analysis

All OS and PFS data from RCTs were analyzed by HR and 95% confidence interval (CI). ORR, the incidence of any grade TRAEs, and grade 3 to 5 TRAEs were assessed by risk ratio (RR) and 95% CI. OS and PFS were further stratified depending on the status of PD-L1 expression. Because the definition of positive expression of PD-L1 varied among clinical trials, we selected PD-L1TPS ≥ 10% or combined positive score (CPS) ≥ 10%, 1% ≤ TPS < 10% or 1% ≤ CPS < 1 0%, TPS < 1% or CPS < 1% which was assayed by immunohistochemistry staining methods, score 1 as a cutoff to identify PD-L1 positive patients in our studies. The *I*^2^ statistic and *Q* test were used to assess the heterogeneity among the studies. The application of fixed-effects (*I*^2^ ≤ 50%) or random-effects (*I*^2^ > 50%) models depended on the degree of trial heterogeneity. The meta-analysis was conducted using Review Manager 5.3 (Cochrane Collaboration, Oxford, UK) and R studio. Statistics were judged significant at *P* < .05. Additionally, the Cochrane Risk of Bias Tool was used to assess the risk of bias. The authors discussed and reached consensus on all differences.

### 2.5. Protocol and ethics

We registered on PROSPERO, and ID number is CRD42023444581. No ethical approval was needed for this meta-analysis.

## 3. Results

### 3.1. Search results and study characteristics

**Figure 1.**
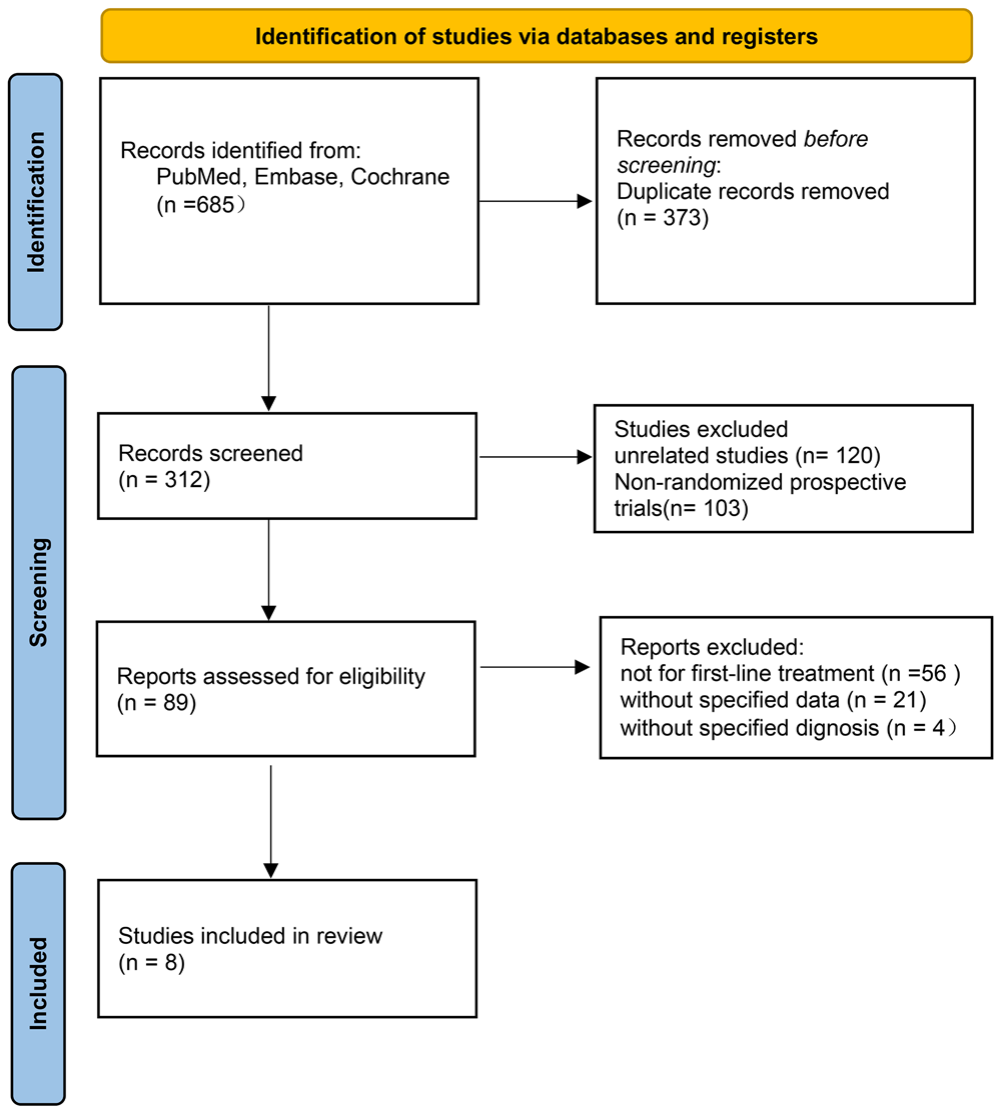
Flow diagram: selection process for the studies. The flowchart presents the number and types of studies included and excluded at each stage of the selection process.

Figure 1 displays the procedure for selecting the literature for this meta-analysis as well as the outcomes of the search. Following screening and eligibility evaluation, our investigation turned up a total of 685 relevant records. 373 reports were eliminated due to duplication, and 304 records were eliminated for various other factors. Eight studies were included in the final analysis, while 17 studies were evaluated for eligibility.

This study comprised a total of 4702 trial participants from the 8 RCTs, 2529 of those received anti-PD-1 along with chemotherapy to be first-line therapy for ESCC, and the remaining 2173 received chemotherapy. Furthermore, 8 clinical trials were published from 2021 to 2024.^[9-16]^ Six multicenter clinical studies were international, and 2 from China. Both studies were multicenter phase III clinical trials. Patients were divided into 3 groups for the checkmate 648 trial: immunochemotherapy, immunotherapy alone, and chemotherapy alone. Both ESCC and EAC patients were included in KEYNOTE-590. We only use ESCC data. Table 1 displays the fundamental elements of the included literature.

**Table 1:** Characteristics of the studies included in the meta-analysis.

Study	Author	Year	Trial phase	Control group						Study group							
				Treatment	n	mOS (months)	mPFS (months)	ORR (%)	TRAE ≥ 3	Treatment	n	mOS (mo)	mPFS (mo)	ORR (%)	HR for OS (95% CI)	HR for PFS (95% CI)	TRAE ≥ 3
ESCORT-1^st^	Luo	2021	III	Placebo-TP	298	12.0 (11.0–13.3)	5.6 (5.5–5.7)	185/298 62.1% (56.3–67.6%)	189 (63.4%)	Camrelizumab-TP	298	15.3 (12.8–17.3)	6.9 (5.8–7.4)	215/298 72.1% (66.7–77.2%)	0.70 (0.56–0.88)	0.56 (0.46–0.68)	201 (67.7%)
CheckMate 648	Doki	2022	III	CF	324	10.7 (9.4–11.9)	5.6 (4.3–5.9)	87/324 27% (22–32%)	108 (36%)	Nivolumab-CF	321	13.2 (11.1–15.7)	5.8 (5.6–7.0)	152/321 47%(42–53%)	0.74 (0.58–0.96)	0.81 (0.64–1.04)	147 (47%)
										Nivolumab-ipilimumab	325	12.7 (11.3–15.5)	2.9 (2.7–4.2)	90/325 28%(23–33%)	0.78 (0.62–0.98)	1.26 (1.04–1.52)	102 (32%)
JUPITER-06	Wang	2022	III	Placebo-TP	257	11 (10.4–12.6)	5.5 (5.2–5.6)	134/257 52.1% (45.8–58.4%)	180 (70.0%)	Toripalimab-TP	257	17.0 (14.0-not reached)	5.7 (5.6–7.0)	178/257 69.3%(63.2–74.8%)	0.58 (0.43–0.78)	0.58 (0.46–0.74)	188 (73.2%)
KEYNOTE-590	Sun	2021	III	Placebo-CF	274	9.8 (8.6–11.1)	5.8 (5.0–6.1)	110/376 29.3% (24.7–34.1%)		Pembrolizumab-CF	274	12.6 (10.2–14.3)	6.3 (6.2–6.9)	168/373 45.0%(39.9–50.2%)	0.72 (0.60–0.88)	0.65 (0.54–0.78)	
ORIENT-15	Lu	2022	III	Placebo-TP/CF	332	12.5 (11.0–14.5)	5.7 (5.5–6.8)	151/332 45% (40–51%)	181 (55%)	Sintilimab-TP/CF	327	16.7 (14.8–21.7)	7.2 (7.0–9.6)	216/327 66%(61–71%)	0.63 (0.51–0.78)	0.56 (0.46–0.68)	196 (60%)
RATIONALE-306	Xu	2023	III	Placebo-CF/TP	323	10.6 (9.3–12.1)	5.6 (4.9–6.0)	137/323 42% (37–48%)	219 (68.22%)	Tislelizumab-CF/TP	326	17.2 (15.8–20.0)	7.3 (6.9–8.3)	207/326 63%(58–69%)	0.66 (0.54–0.80)	0.62 (0.52–0.75)	224 (70%)
ASTRUM-007	Song	2023	III	Placebo-CF	183	11.8 (9.7–14.0 )	5.3 (4.3–5.6)	42.1% (34.8–49.6%)	81 (48%)	Serplulimab-CF	368	15.3 (14.0–18.6)	5.8 (5.7–6.9)	57.6% (52.4–62.7%)	0.68 (0.53–0.87)	0.60 (0.48–0.75)	201 (53%)
GEMSTONE-304	Li	2023	III	Placebo-CF	182	11.5 (9.9–13.4)	5.4 (4.9–5.8)	45.2% (37.7–52.8%)	88 (48.4%)	sugemalimab-CF	358	15.3 (13.3–17.1)	6.2 (5.7–6.9)	60.1% (54.7–65.2%)	0.7 (0.55–0.9)	0.67 (0.54–0.82)	181 (51.3%)

CF = 5-fluorouracil+cisplatin, HR = hazard ratio, mOS = median overall survival, mPFS = median progression-free survival, ORR = objective response rate, OS = overall survival, PFS = progression-free survival, TP = platinum+taxanes, TRAE = treatment-related adverse event.

### 3.2. Outcome indicator

**Figure 2.**
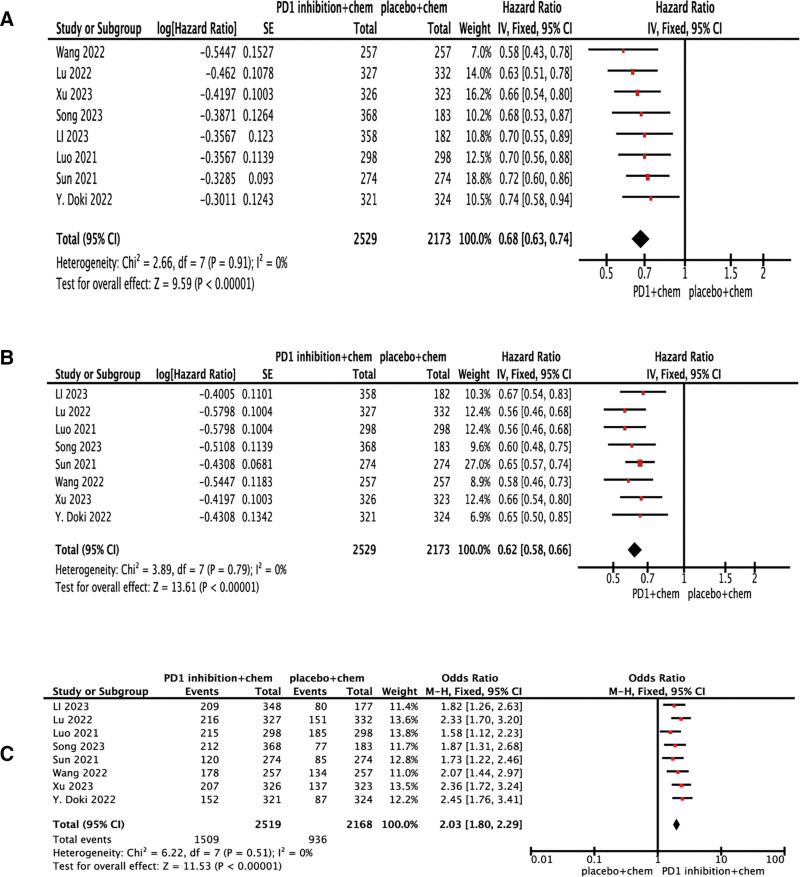
Forest plots for outcomes in patients treated with PD-1 inhibitors versus chemotherapy: (A) OS (B) PFS (C) ORR. CI = confidence interval, IV = inverse variance, ORR = objective response rate, OS = overall survival, PFS = progression-free survival, SE = standard error.

Pooled data from 8 studies (n = 2529 patients receiving PD-1 inhibitors plus chemotherapy; n = 2173 receiving placebo plus chemotherapy) demonstrated superior efficacy for PD-1 inhibitor combination therapy across all reported endpoints: OS, PFS, and ORR. Results revealed that PD-1 inhibitors outperformed chemotherapy in terms of OS (HR: 0.68, 95% CI: 0.63–0.74, *P* < .00001; heterogeneity: *I*^2^ = 0.0%, *P* = .91). Compared with standard chemotherapy, PD-1 inhibitors plus chemotherapy also performed significantly favorable in term of PFS (HR: 0.62, 95% CI: 0.58–0.66, *P* < .00001; heterogeneity: *I*^2^ = 0.0%, *P* = .74). ORR was also superior in the PD-1 inhibitor group to the chemotherapy group in first-line (OR: 2.03, 95% CI: 1.80–2.29, *P* < .00001; Fig. 2).

**Figure 3.**
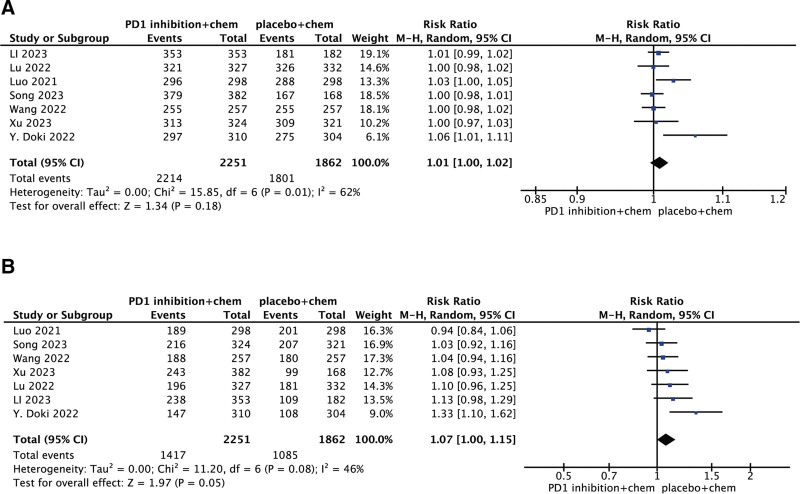
Forest plots of RR for TRAEs (A) and grade 3 to 5 TRAES (B) in patients treated with PD-1 inhibitors plus chemotherapy versus chemotherapy alone. CI = confidence interval, PD-1 = programmed death-1, RR = relative risk, TRAE = treatment-related adverse event.

Eight studies reported any-grade TRAEs demonstrating no comparable incidence between the intervention and control groups (RR: 1.01, 95% CI: 1.00–1.02; *P* = .01; *I*^2^ = 64%; Fig. 3). Also, there was no significant difference in grade 3 or higher TRAE (RR: 1.07, 95% CI: 1.00–1.15; *P* = .05; *I*^2^ = 46%). Therefore, the addition of PD-1 inhibitors did not increase the incidence of adverse events (AEs) or treatment-related adverse events (TRAEs), demonstrating that PD-1 inhibitor combination chemotherapy is safe with reliable clinical profiles.

### 3.3. OS and PFS in younger and older patients

**Figure 4.**
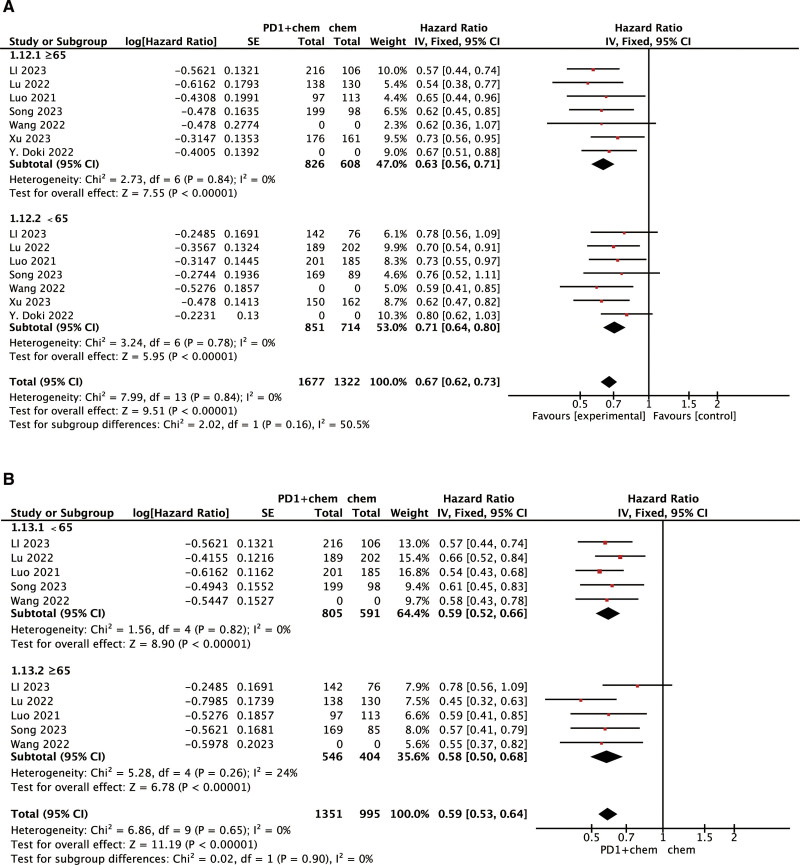
Subgroup analysis of the effects of age for OS (A) and PFS (B). CI = confidence interval, OS = overall survival, PD-1 = programmed death-1, PFS = progression-free survival.

It has been reported that age also affects treatment efficacy during PD-1 inhibitor therapy.^[17,18]^ Therefore, we performed an age-stratified subgroup analysis (≥65 vs <65 years). As shown in Figure 4, PD-1 inhibitors also significantly improved OS (HR, 0.63; 95% CI: 0.56–0.71) and PFS (HR, 0.58; 95% CI: 0.50–0.68) in patients aged ≥65.

### 3.4. OS and PFS in different PD-L1 expression status

**Figure 5.**
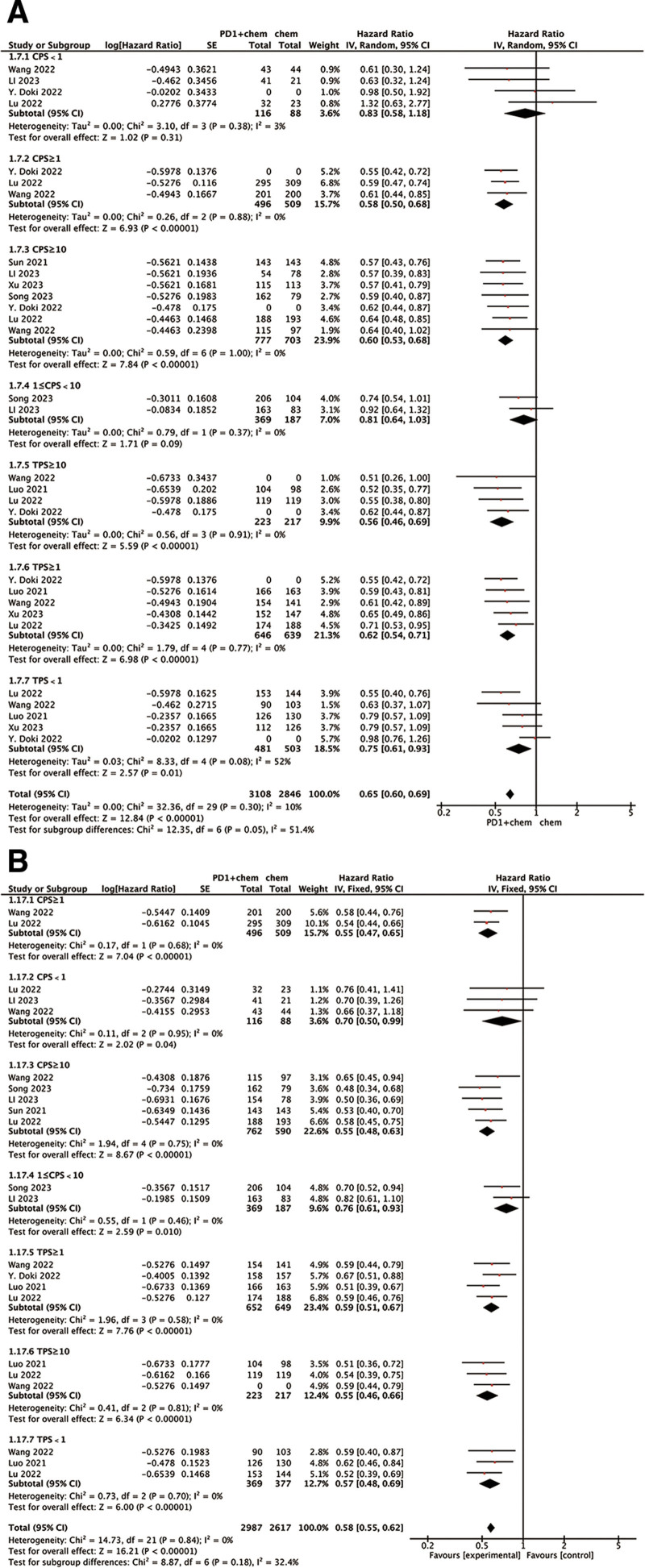
Subgroup analysis of HR for OS (A) and PFS (B) in the patients with different status of PD-L1 expression assigned to the PD-1 inhibitor plus chemotherapy group, compared with those in the chemotherapy group. CI = confidence interval, CPS = combined positive score, OS = overall survival, PD-1 = programmed death-1, PD-L1 = programmed death-ligand 1, PFS = progression-free survival, SE = standard error, TPS = tumor proportion score.

We also analyzed OS and PFS in different PD-L1 status subgroups. TPS and CPS are most commonly used PD-L1 scoring methods.^[19-22]^ The HR for OS and PFS favored PD-1 inhibitor-based therapy over chemotherapy in high PD1 (CPS ≥ 1, TPS ≥ 1) expression subgroups, but the OS and PFS benefit was limited in the PD-L1 TPS < 1 subgroups, and there is no OS significant benefit in the subgroup of PD-L1 CPS < 1 (HR: 0.83, *P* = .31; Fig. 5). Particularly, patients with TPS ≥ 10 or CPS ≥ 10 demonstrated more pronounced clinical benefits from anti-PD-1-based therapy.

### 3.5. OS and PFS based on disease status

**Figure 6.**
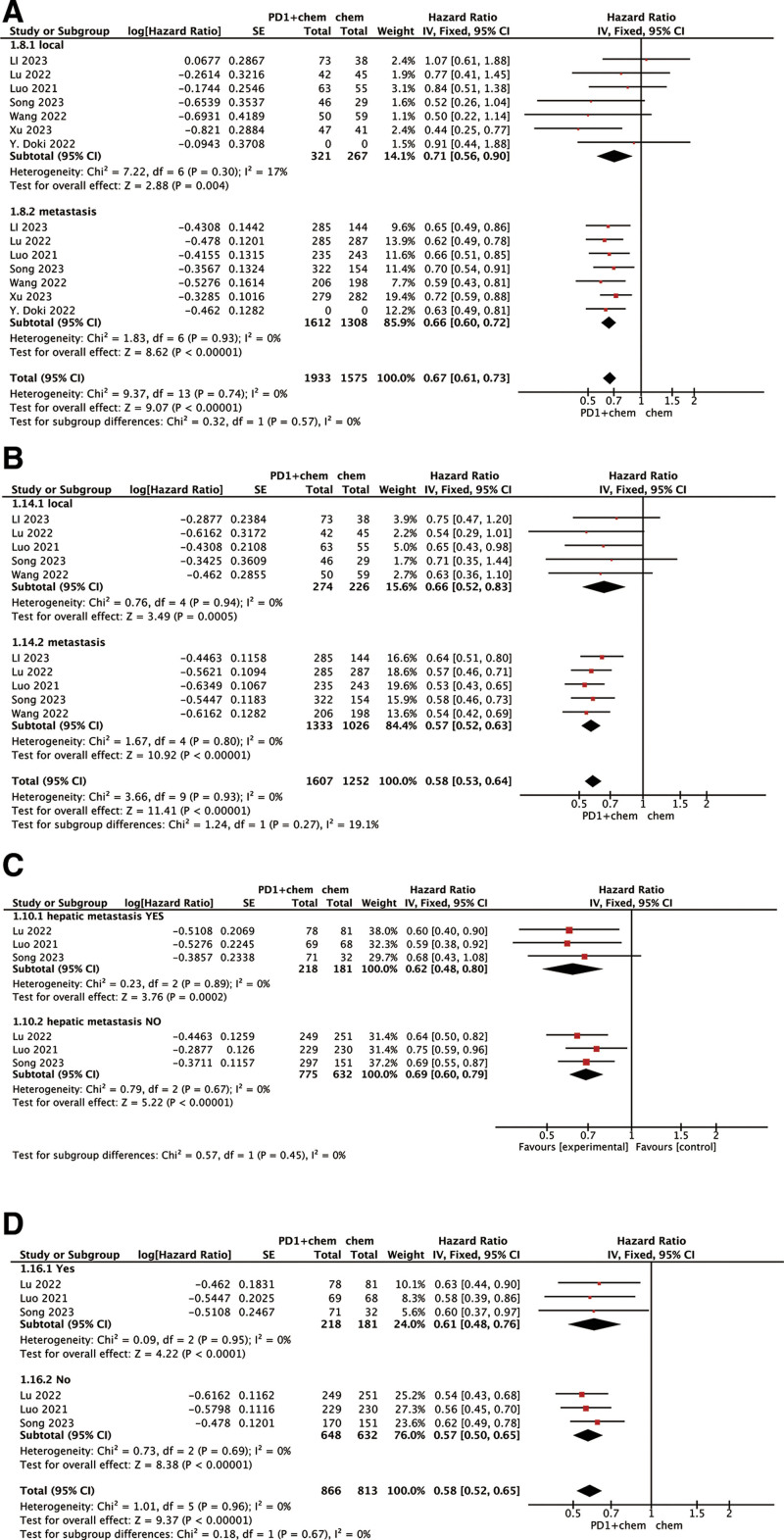
Subgroup analysis of HR for OS (A) and PFS (B) in the patients presence or absence of distant metastasis s assigned to the PD-1 inhibitor plus chemotherapy group, compared with those in the chemotherapy group. Subgroup analysis of the effects for OS (C) and PFS (D) in the patients with or without hepatic metastasis. CI = confidence interval, OS = overall survival, PD-1 = programmed death-1, PFS = progression-free survival, SE = standard error.

We also performed a subgroup analysis based on disease status. PD-1 inhibitors demonstrated clinical benefit in advanced patients with metastases, significantly extending both OS (HR, 0.66; 95% CI: 0.60–0.72) and PFS (HR, 0.57; 95% CI: 0.52–0.63) in the metastatic subgroup. Additionally, some researchers consider that the tumor microenvironment (TME) in liver metastases (LM) exhibits low immune activation, leading to suboptimal organ-specific responses to immunotherapy.^[23]^ Therefore, we conducted a subgroup analysis of patients with liver metastases to evaluate PD-1 inhibitor efficacy. Results indicated no comparable clinical outcomes between ESCC patients with and without liver metastases (Fig. 6).

### 3.6. Sensitivity analyses and publication bias

**Figure 7.**
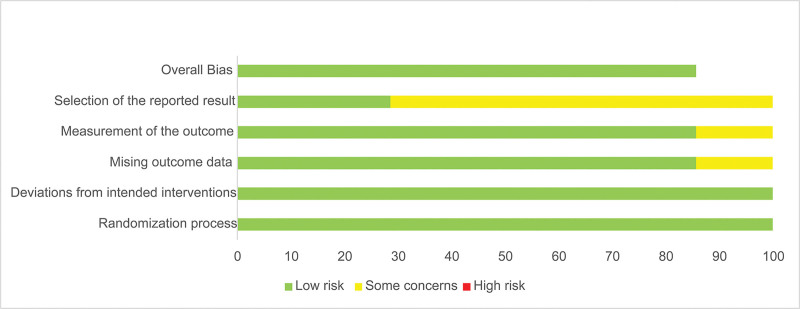
Quality evaluation of included studies.

Risk of bias assessment for included studies indicated high methodological quality, as all were phase III RCTs. Publication bias evaluation was precluded due to the limited number of studies (<10; Fig. 7).

## 4. Discussion

In recent years, PD-1/PD-L1 immune checkpoint inhibitors combined with chemotherapy have become the standard first-line therapy for advanced EC, significantly improving survival outcomes. The advent of immunotherapy, particularly its combination with chemotherapy, has demonstrated substantial enhancement in both OS and PFS. Both PD-1 and PD-L1 inhibitors typically achieve improvement in efficacy metrics (ORR/PFS). This combination regimen now constitutes the cornerstone of first-line treatment. Notably, the integration of immunotherapy with chemotherapy or radiotherapy transcends additive effects (“1 + 1=2”), instead exhibiting synergistic enhancement (“1 + 1>2”) through mutually reinforcing mechanisms.^[8,24]^

Regional variations persist, with some areas favoring platinum plus fluoropyrimidine-based regimens, while others prefer platinum combined with taxanes. Both the ORIENT-15 and RATIONALE-306 trials incorporated these distinct chemotherapy backbones, yet demonstrated comparable efficacy when combined with PD-1 inhibitors.

No global consensus exists regarding the optimal first-line chemotherapy regimen for patients with advanced or metastatic ESCC. Liver metastasis is an independent predictor of poor prognosis in ESCC.^[25]^ Although historically associated with reduced immunotherapy response, subgroup analyses from 3 pivotal phase III trials – ASTRUM-007, ESCORT-1st, and ORIENT-15 demonstrated significant survival benefits with PD-1 inhibitor plus chemotherapy combinations in this high-risk population. Furthermore, PD-1 inhibitor-based combination chemotherapy consistently achieved clinically meaningful survival outcomes in ESCC patients with both oligometastatic and polymetastatic disease.

ORIENT-15 used 2 main scoring algorithms, tumor proportion score (TPS) and CPS. CPS may be more likely than TPS to identify a greater proportion of patients who may benefit from anti-PD-1 therapy and was therefore used in the present trial for patient screening. In the JUPITER-06 and KEYNOTE-590 trials, PD-L1 expression assessment uniformly employed the JS311 antibody. Analytical comparability studies with 22C3 and 28-8 antibodies across multiple tumor types – including melanoma, urothelial carcinoma, ESCC, and non-small cell lung cancer (NSCLC) demonstrated an overall concordance rate of 80% to 90% in immunohistochemical staining results.^[22,23]^ This result revealed no significant clinical benefit in patients with PD-L1 expression < 1%. However, PFS demonstrated more pronounced improvement than OS, supporting the NCCN guideline recommendation of PD-L1 expression clinical benefit in patients with PD-L1 expression < 1%.^[26,27]^

Our meta-analysis has the following advantages. All included studies were large phase 3 clinical trials, and all data were the latest, indicating that the results of this meta-analysis are reliable. We carried out detailed subgroup analyses according to PD1 expression, age, and metastasis results will be greatly useful in Clinical decision-making. This study had some limitations, like the number of retrieved studies was relatively small, as only 8 RCTs were included, and patient enrollment was predominantly within mainland China. We need to collect more data from outside China. In addition, subgroup analyses according to PD-L1 testing methods are not available because of the limitation in the number of clinical trials. Attention to such progress in advanced ESCC is still needed.

## 5. Conclusion

In conclusion, this study is the latest meta-analysis to systematically review the clinical efficacy and therapeutic safety of PD-1 inhibitors in patients with advanced ESCC in the first-line setting. PD-1 inhibitors plus chemotherapy possessed better OS and safety compared to chemotherapy in advanced ESCC.

## Author contributions

**Conceptualization:** Dong Wang, Xiangyu Zhang.

**Data curation:** Lili Su.

**Funding acquisition:** Rui Gao.

**Project administration:** Tingting Dai.

**Project administration:** Tingting Dai.

**Supervision:** Dong Wang.

**Writing – original draft:** Rui Gao, Tingting Dai.

**Writing – review & editing:** Lili Su.
